# Antitumour 2-(4-aminophenyl)benzothiazoles generate DNA adducts in sensitive tumour cells *in vitro* and *in vivo*

**DOI:** 10.1038/sj.bjc.6600719

**Published:** 2003-02-10

**Authors:** C-O Leong, M Gaskell, E A Martin, R T Heydon, P B Farmer, M C Bibby, P A Cooper, J A Double, T D Bradshaw, M F G Stevens

**Affiliations:** 1 School of Pharmaceutical Sciences, University of Nottingham, Nottingham NG7 2RD, UK; 2 Biocentre, University of Leicester, Leicester LE1 7RH, UK; 3 Cancer Research Unit, School of Life Sciences, University of Bradford, Bradford BD7 1DP, UK

**Keywords:** 2-(4-aminophenyl)benzothiazole, Phortress, breast cancer, ovarian cancer, DNA adducts

## Abstract

2-(4-Aminophenyl)benzothiazoles represent a potent and highly selective class of antitumour agent. *In vitro*, sensitive carcinoma cells deplete 2-(4-aminophenyl)benzothiazoles from nutrient media; cytochrome P450 1A1 activity, critical for execution of antitumour activity, and protein expression are powerfully induced. 2-(4-Amino-3-methylphenyl)benzothiazole-derived covalent binding to cytochrome P450 1A1 is reduced by glutathione, suggesting 1A1-dependent production of a reactive electrophilic species. *In vitro*, 2-(4-aminophenyl)benzothiazole-generated DNA adducts form in sensitive tumour cells only. At concentrations >100 nM, adducts were detected in DNA of MCF-7 cells treated with 2-(4-amino-3-methylphenyl)-5-fluorobenzothiazole (5F 203). 5F 203 (1 *μ*M) led to the formation of one major and a number of minor adducts. However, treatment of cells with 10 *μ*M 5F 203 resulted in the emergence of a new dominant adduct. Adducts accumulated steadily within DNA of MCF-7 cells exposed to 1 *μ*M 5F 203 between 2 and 24 h. Concentrations of the lysylamide prodrug of 5F 203 (Phortress) ≥100 nM generated adducts in the DNA of sensitive MCF-7 and IGROV-1 ovarian cells. At 1 *μ*M, one major Phortress-derived DNA adduct was detected in these two sensitive phenotypes; 10 *μ*M Phortress led to the emergence of an additional major adduct detected in the DNA of MCF-7 cells. Inherently resistant MDA-MB-435 breast carcinoma cells incurred no DNA damage upon exposure to Phortress (⩽10 *μ*M, 24 h). *In vivo*, DNA adducts accumulated within sensitive ovarian IGROV-1 and breast MCF-7 xenografts 24 h after treatment of mice with Phortress (20 mg kg^−1^). Moreover, Phortress-derived DNA adduct generation distinguished sensitive MCF-7 tumours from inherently resistant MDA-MB-435 xenografts implanted in opposite flanks of the same mouse.

Novel 2-(4-aminophenyl)benzothiazoles possess remarkably selective antitumour properties (Shi *et al*, 1996; Bradshaw *et al*, 1998a,1998b) and represent a mechanistic class distinct from clinically used chemotherapeutic agents. Consistently, these molecules are exquisitely potent (GI_50_<10 nM; GI is the drug concentration that inhibits cell growth by 50%) against a specific subset of human cancer cell lines in the National Cancer Institute (NCI) *in vitro* anticancer drug screen, producing highly characteristic mean graph patterns.

2-(4-Amino-3-methylphenyl)benzothiazole (DF 203, NSC 674495) and 2-(4-amino-3-methylphenyl)-5-fluorobenzothiazole (5F 203, NSC 703786) are efficiently sequestered by sensitive cell lines (e.g. breast MCF-7, MDA-468; renal TK-10) (Chua *et al*, 1999a,1999b; Kashiyama *et al*, 1999). In stark contrast, insensitive cell lines (GI_50_>10 *μ*M, e.g. breast MDA-MB-435; renal A498, CAKI-1; prostate PC-3) fail to deplete these agents from nutrient media.

Planar, hydrophobic aminophenylbenzothiazole analogues are potent agonists for the aryl hydrocarbon receptor (AhR). Nuclear translocation follows high affinity (nM) binding IC_50_ between ligand and cytosolic AhR. Xenobiotic response element (XRE)-driven luciferase activity is induced and protein–DNA complexes are formed on the XRE sequence of the cytochrome P450 (CYP) 1A1 promoter (Loaiza-Perez *et al*, 2002). Thus, CYP1A1 mRNA activity and protein expression are induced exclusively in sensitive cell lines (Chua *et al*, 2000).

Crucially, induction of CYP1A1-catalysed biotransformation of 2-(4-aminophenyl)benzothiazoles within tumour cells is essential for drug activation. Covalent binding, detected between DF 203 and recombinant CYP1A1, requires metabolism and is significantly reduced by glutathione, suggesting the formation of an electrophilic, reactive intermediate species. Paradoxically, the C-6 hydroxylated biotransformation product, liberated into nutrient media of cells exposed to DF 203, is devoid of antitumour activity; moreover, this metabolite is able to antagonise cellular uptake of DF 203, covalent binding between CYP1A1 and DF 203, CYP1A1 activity and growth inhibition induced by DF 203 (Hutchinson *et al*, 2001).

Using *ab initio* frontier molecular orbital (FMO) calculations, the presence or absence of exportable hydroxy metabolites of fluorinated 2-(4-amino-3-methylphenyl)benzothiazoles can be predicted (O'Brien *et al*, in press). Only the 5-fluoro isomer produced a conventional dose–response following treatment of cells; indeed, oxidative metabolism at C-6 was eradicated. Thus, 2-(4-amino-3-methylphenyl)-5-fluorobenzothiazole (5F 203, NSC 703786) is the favoured analogue for clinical consideration possessing enhanced efficacy *in vitro* and superior potency *in vivo* against human breast and ovarian tumour xenografts implanted in nude mice (Hutchinson *et al*, 2001; Bradshaw *et al*, 2002a). The lysylamide dihydrochloride salt of 5F 203 (Phortress, NSC 710305) has been derivatised at the exocyclic primary amine function fulfilling the criteria for a suitable prodrug. It is water soluble, chemically stable, pharmaceutically robust and undergoes rapid, quantitative bioreversion to the parent moiety *in vitro* and *in vivo* (Bradshaw *et al*, 2002a,2002b; Hutchinson *et al*, 2002) (Figure 1

**Figure 1:**
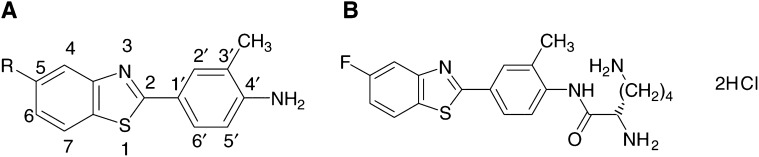
Structures of (**A**) 2-(4-amino-3-methylphenyl)benzothiazole (R=H; DF 203; NSC 674495), 2-(4-amino-3-methylphenyl)-5-fluorobenzothiazole (R=F; 5F 203, NSC 703786) and (**B**) the lysylamide dihydrochloride salt of 5F 203 (Phortress, NSC 710305).

).

Additionally, a putative, as yet undefined, role of CYP1B1 in the mechanism of action of aminophenylbenzothiazoles has been implicated. CYP1B1 microsomes are able to metabolise DF 203, and modulation of CYP1B1 expression in sensitive cell lines has been observed in response to benzothiazole treatment as well as induction of CYP1B1 mRNA in MCF-7 cells. The CYPs comprise a superfamily of haemoproteins that catalyse the initial monooxygenation of a range of lipophilic endogenous, environmental or dietary xenobiotic substrates (Guengerich, 1992; Crofts *et al*, 1998). The substrate specificities of CYP1A1 and CYP1B1 overlap: both isoforms are important in the metabolism of polycyclic aromatic hydrocarbons (PAHs) and some heterocyclic amines (Shimada *et al*, 1996,^1998^).

We have examined the hypothesis that exposure to analogues of this unique class of antitumour agent selectively generates benzothiazole-derived covalent DNA adducts in sensitive cell lines. The powerful ^32^P-postlabelling technique has been adopted for the detection of DNA adducts (Martin *et al*, 1997,^1998^).

Optimisation of the procedure for detection and separation of benzothiazole-DNA adducts preceded *in vitro* studies to investigate time- and dose-dependent accumulation of 5F 203-DNA adducts in MCF-7 mammary carcinoma cells. 5F 203-derived adducts have been compared with DNA adduct formation in MCF-7 and IGROV-1 ovarian carcinoma cells following treatment with Phortress. We demonstrate selective generation of adducts in the DNA of sensitive tumour cells *in vitro* and disclose chromatographically equivalent adduct formation *in vivo* in MCF-7 and IGROV-1 xenografts, following treatment of mice, i.p. with Phortress (20 mg kg^−1^).

## MATERIALS AND METHODS

### Cell culture

Human-derived carcinoma cell lines (MCF-7, MDA-MB-435 breast, HCT 116 colon and IGROV-1 ovarian) were cultured in RPMI-1640 medium supplemented with 10% FBS. Cells were subcultivated twice weekly to maintain logarithmic growth. Amines were prepared in DMSO as a 10 mM stock. Phortress was prepared in medium immediately prior to use. Cells were seeded at the appropriate density and, after 24 h, nutrient media refreshed and drug introduced. Following the desired exposure period, cells were harvested by trypsinisation, washed in PBS and counted.

### *In vivo* procedure

UKCCCR guidelines for the welfare of animals in experimental neoplasia were adhered to during all *in vivo* studies. MCF-7, MDA-MB-435 breast and IGROV-1 ovarian xenografts were transplanted s.c. into flanks of NCR-Nu female nude mice. Animals were treated i.p. with 20 mg kg^−1^ Phortress (*n*=4) or vehicle alone (saline). Pellets (60 day release 17*β*-estradiol, 0.72 mg pellet^−1^: Innovative Research, USA) were implanted s.c. in mice bearing MCF-7 xenografts maintaining blood levels of 300–400 pg ml^−1^. Following 24 h exposure, tumours were removed and snap frozen (−80°C). To examine the selective nature of DNA adduct formation *in vivo*, mice bearing MCF-7 and MDA-MB-435 tumours in opposite flanks were treated with Phortress. After 24 h treatment, animals were killed. Tumours were recovered and snap frozen.

### Determination of DNA adducts

DNA was extracted from cells or xenograft tissue using Qiagen DNA extraction columns (Qiagen, Crawley, UK) as described in the manufacturer's instructions. DNA was dissolved in 0.5–1 ml of 1 : 100 SSC (0.015 M sodium citrate, 0.15 M NaCl, pH 7.2) and absorbance recorded at 230, 260, 280 and 320 nm using a UV spectrometer. The quantity of DNA was calculated from the absorbance at 260 nm assuming that an absorbance value of 1 is equivalent to a DNA concentration of 50 *μ*g ml^−1^. The purity of the extracted DNA was determined from the 260/280 nm absorbance ratio. DNA with a ratio >1.7 was considered pure.

DNA was dried and hydrolysed to deoxynucleoside 3′-monophosphates by incubating 5 *μ*g of DNA with micrococcal nuclease (175 mU), calf-spleen phosphodiesterase (3 mU) in 1 *μ*l of SSCC (100 mM sodium succinate, pH 6.0: 50 mM CaCl_2_) and d-H_2_O to a total volume of 6.25 *μ*l. The mixture was incubated at 37°C overnight.

Adducts were enriched by butanol extraction. Digested DNA (5 *μ*g) was diluted with 115 *μ*l HPLC-grade water. To each sample, 100 mM ammonium formate (15 *μ*l, pH 3.5) and 10 mM tetrabutylammonium chloride (TBAC) (15 *μ*l) were added. The mixture was extracted twice with water saturated 1-butanol. The combined organic phase was back-extracted twice with 250 *μ*l×butanol saturated water to remove trace contaminants of normal nucleotides. The butanol extract was then neutralised by the addition of 3 *μ*l 200 mM Tris HCl (pH 7.6) and evaporated in a Speed Vac Concentrator (Savant Instruments, Inc., Hickville, NY, USA). Radiolabelling was achieved by incubating adducted nucleotides from 5 *μ*g DNA with T4 polynucleotide (6.25 units) and 62.5 *μ*Ci [*γ*^32^P]ATP for 1 h at 37°C.

The ^32^P-labelled products were diluted to 100 *μ*l with HPLC-grade water. The solution was injected onto a Hypersil ODS C18 analytical column (250×4.6 mm, 5 *μ*M; Shandon), eluted at a flow rate of 1.0 ml min^−1^ with 2 M ammonium formate (pH 4.0) (A) and acetonitrile (B). HPLC gradient: 88% (v v^−1^) A initial mixture, 50 min linear gradient to 75% A followed by 15 min linear gradient to 55% A. The radioactivity was monitored by a radiochemical detector (Lab Logic, *β*-RAM, Sheffield) lined to a Varian Star 9012 pump. Data analysis was by Laura (MS Windows package, Lab Logic Inc, Sheffield).

For quantification of the adduct levels, known amounts of [γ-^32^p] ATP were injected onto the HPLC, peaks were collected and radioactivity was measured in disintegrations per min (dpm) by scintillation counting. ATP peak area was plotted against dpm to give a standard curve. For subsequent analyses, HPLC peak areas were measured and values were applied to this standard curve to give values in dpm. Relative adduct levels (RAL) were calculated according to Reddy and Randerath (1986) and translated into fmol adducts per *μ*g DNA by multiplying RAL×10^7^×0.3240 (Gupta, 1985). The specific activity of [*γ*-^32^P]ATP was corrected by calculating the extent of decay.

Preliminary studies included enrichment of adducts by nuclease P1-catalysed dephosphorylation, exploiting the ability of certain bulky adducts to protect the nucleotide from digestion by nuclease P1 and the absolute requirement of the 3′ phosphate in the subsequent labelling step (transfer of *γ*-^32^P from ^32^P-ATP to the 5′ position of the nucleotides by the enzyme T4 polynucleotide kinase to give 3′, 5′-bisphosphates). In addition to HPLC analyses, adducted DNA was separated by thin layer chromatography (TLC) in two directions on 10×10 cm PEI-cellulose TLC plates. Samples were spotted in the lower left corner of each plate and run from top to bottom in 2.3 M sodium phosphate, pH 5.8, onto an attached wick. After washing and drying, the plates were developed from bottom to top in 2.79 M lithium formate, 4.95 M urea, pH 3.4. The final chromatographic washes were run at 90° (left to right) in 0.75 M sodium dihydrogenhosphate, 0.45 M Tris, 8.0 M urea, pH 8.2 and then 1.7 M sodium phosphate, pH 6.0 onto an attached wick.

## RESULTS

### *In vitro*

**Figure 2:**
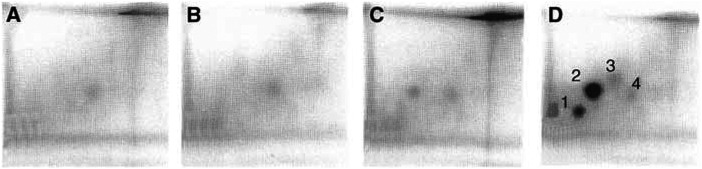
TLC phosphorimager images of ^32^P-postlabelled DNA from MCF-7 (**B** and **D**) and HCT 116 (**A** and **C**) cell lines, untreated (**A** and **B**) and following exposure to DF 203 (1 *μ*M 72 h, **C** and **D**).

Preliminary studies clearly demonstrated DNA adduct formation in sensitive cells only (e.g. MCF-7, MDA 468 human mammary carcinoma cell lines) following their exposure to DF 203, irrespective of the analytical method adopted (Stevens *et al*, 2001). Enrichment of adducts by butanol extraction or nuclease P1 digestion prior to adduct separation by TLC (Figure 2

) or HPLC unequivocally demonstrated the formation of one major and a number of minor adducts in the DNA of MCF-7 cells after drug treatment (1 *μ*M DF 203, 72 h). Insensitive HCT 116 cells incurred negligible DNA damage. The chromatographic profile of DNA adducts in MDA 468 cells was identical to that generated in MCF-7 cells (result not shown). No adducts were detected in HPLC traces of untreated cells.

It was concluded that butanol extraction prior to quantitative HPLC separation of benzothiazole-derived adducts was the protocol most suitable for their detection using the ^32^P-postlabelling technique.

### 5F 203 dose response

**Figure 3:**
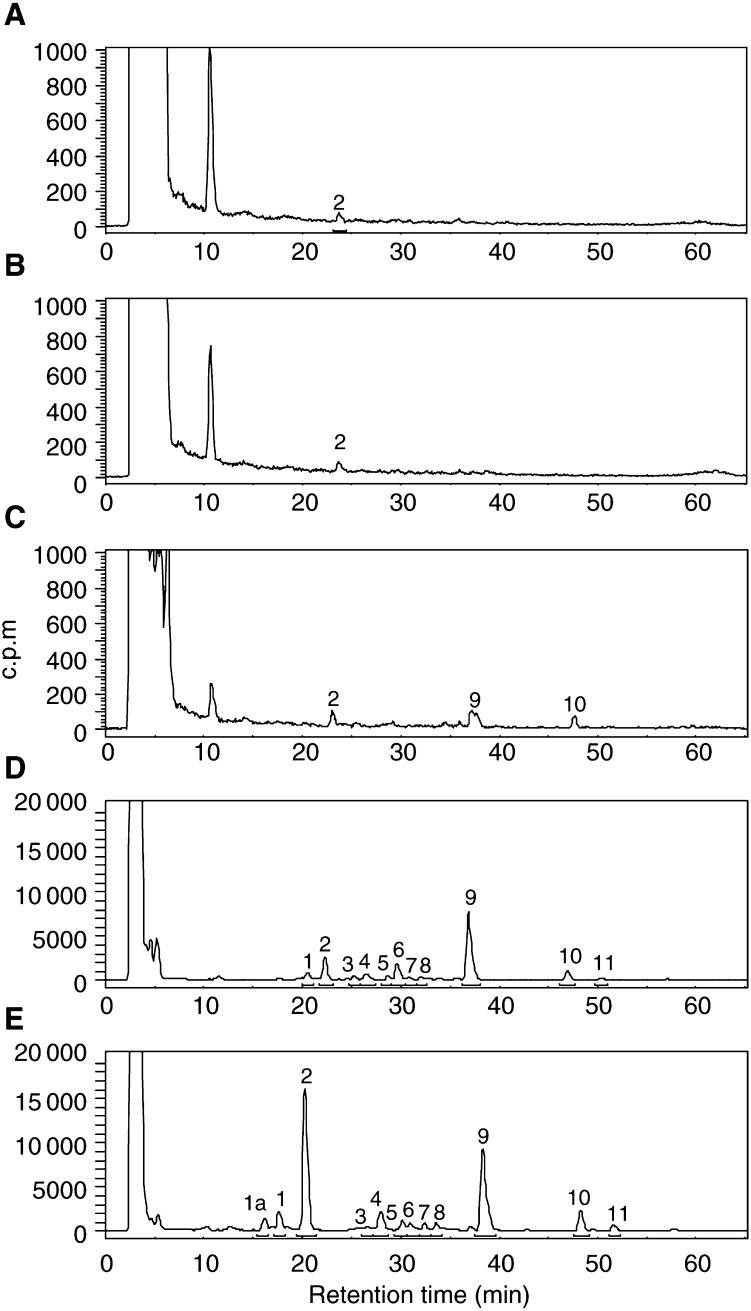
Radiochromatograms of ^32^P-postlabelled DNA adducts in DNA of MCF-7 cells following incubation with (**A**) medium alone, (**B**) 10 nM, (**C**) 100 nM, (**D**) 1 *μ*M and (**E**) 10 *μ*M 5F 203 for 72 h. Adducts were enriched by butanol extraction prior to postlabelling and separated by HPLC. Note the *y* scale of chromatograms for control, 10 and 100 nM is 1000 c.p.m. while the scale for 1 and 10 *μ*M is 20 000 c.p.m. to aid resolution of the adduct profile.

MCF-7 mammary carcinoma cells were exposed to concentrations of 5F 203 between 10 nM and 10 *μ*M for 72 h before DNA was extracted, adducted nucleotides enriched and adduct levels determined by HPLC (Figure 3

). Following exposure of cells to 10 nM 5F 203, very few adducts were detected. 5F 203-derived DNA adducts (labelled **2**, **9** and **10**) were separated after treatment of cells with 100 nM drug. 1 *μ*M 5F 203 effected considerable adduct formation; 1 major adduct, **9**, and a number of minor adducts (e.g. represented by peaks **2** and **10**) were evident. Following treatment of cells with 10 *μ*M 5F 203, the total number of adducts increased; strikingly, the major adduct had become adduct **2** (Figure 3E). Adducts (represented by peak **9**) increased only slightly.

### Accumulation of DNA adducts with time

**Figure 4:**
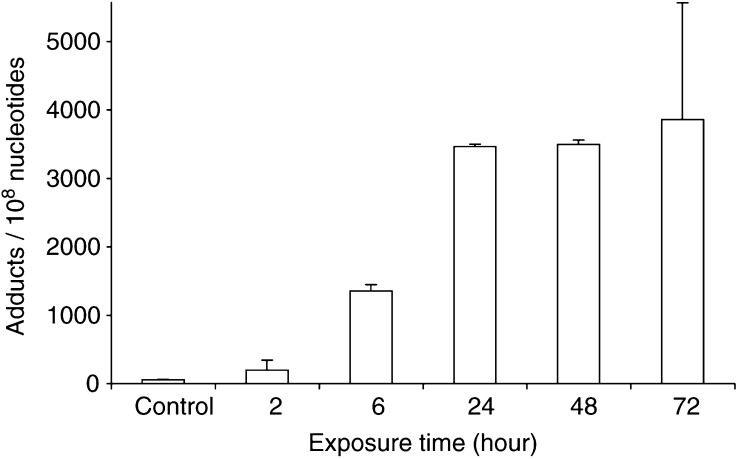
Accumulation of DNA adducts in MCF-7 cells *in vitro*, treated with 1 *μ*M 5F 203. Following extraction of DNA, adducts were enriched by butanol extraction, ^32^P-postlabelled and separated by HPLC. Bars, s.d. (*n*⩾3).

After only 2 h treatment (1 *μ*M 5F 203) adducts could be detected in the DNA of MCF-7 cells. Accumulation of DNA adducts was evident up to 24 h exposure, at which point potential for further adduct formation appeared saturated (Figure 4

).

### Selective generation of Phortress-derived DNA adducts *in vitro*

**Figure 5:**
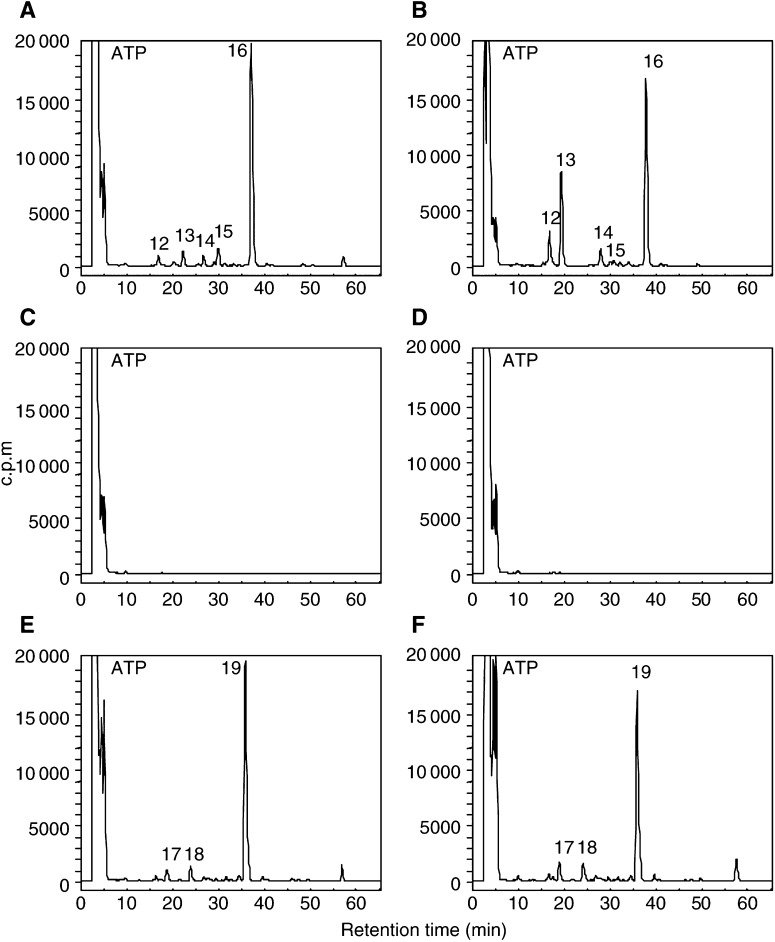
HPLC separation of butanol-extracted, ^32^P-postlabelled DNA adducts in MCF-7 (**A** and **B**), MDA-MB-435 (**C** and **D**) and IGROV-1 (**E** and **F**) cells following exposure to 1 *μ*M (**A**, **C** and **E**) and 10 *μ*M Phortress (**B**, **D** and **F**) *in vitro*. MCF-7 and MDA-MB-435 cells were exposed to drug for 72 h. DNA adducts generated in IGROV-1 cells were analysed after 24 h treatment.

Adducts were detected in the DNA of MCF-7 and IGROV-1 cells exposed to concentrations of Phortress ⩾100 nM: 1 *μ*M Phortress induced one major and a number of minor adducts (Figure 5

). However, adduct profiles in these two cell lines were not identical: peaks equivalent to adducts **14** and **15** in the DNA of MCF-7 cells were absent in DNA extracted from IGROV-1 cells exposed to 1 *μ*M Phortress. MCF-7 cells received 72 h Phortress exposure, whereas after this length of time Phortress was lethal to IGROV-1 cells. Thus, DNA adduct accumulation was analysed in IGROV-1 cells after 24 h exposure to Phortress. The profile and number of DNA adducts in DNA isolated from IGROV-1 cells do not differ significantly whether exposed to 1 or 10 *μ*M Phortress. However, following treatment of MCF-7 cells with 10 *μ*M Phortress, a new major adduct emerged (peak **13**). Significantly, the DNA of MDA-MB-435 cells treated with Phortress (72 h, 10 nM–10 *μ*M) remained free from Phortress-derived adducts.

### Generation of Phortress-derived DNA adducts *in vivo*

**Figure 6:**
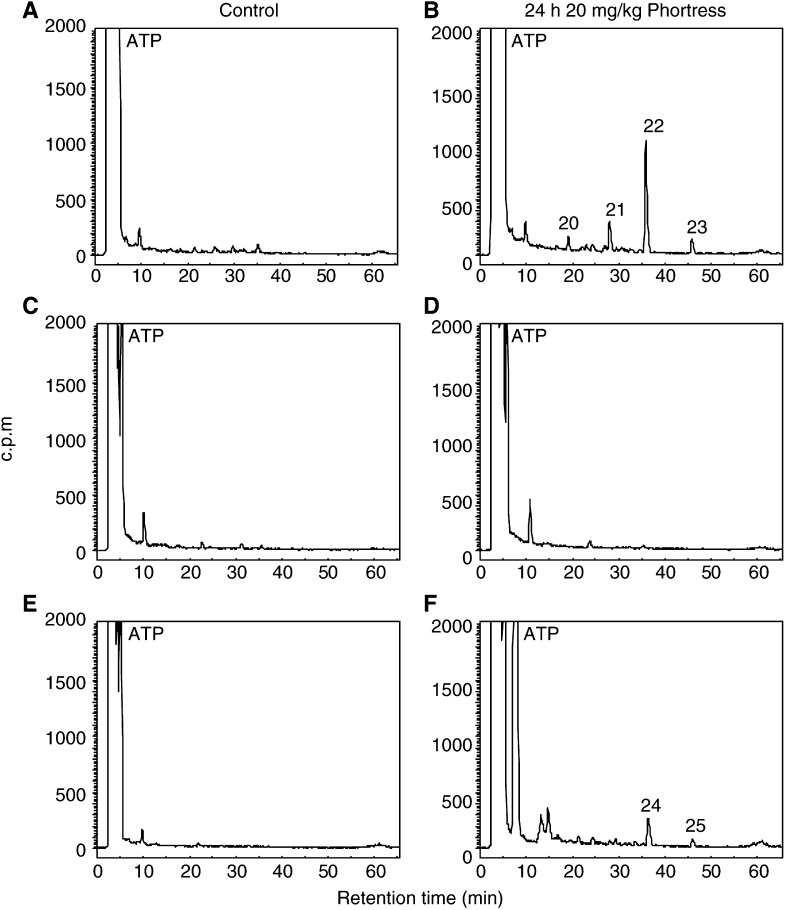
HPLC separation of ^32^P-postlabelled adducts of control MCF-7 (**A**), MDA-MB-435 (**C**) and IGROV-1 (**E**) xenografts from untreated animals and MCF-7 (**B**), MDA-MB-435 (**D**) and IGROV-1 (**F**) xenografts from mice treated with 20 mg kg^−1^ Phortress i.p. Tumours were recovered 24 h post-treatment.

Mice bearing sensitive MCF-7, IGROV-1 and inherently resistant MDA-MB-435 tumours s.c. in the flank were treated with 20 mg kg^−1^ Phortress or vehicle alone (i.p.). Guided by *in vitro* data (Figure 4) and the knowledge that CYP1A1 protein can clearly be detected within sensitive xenografts 24 h post-treatment (Bradshaw *et al*, 2002a), tumours were recovered 24 h after animals were treated and DNA extracted. HPLC detection of ^32^P-postlabelled adducts clearly distinguished one major and a number of minor Phortress-derived adducts in the DNA of MCF-7 xenografts (Figure 6

). As the technique is quantitative, it can be seen that the number and diversity of adducts sustained in IGROV-1 tumours is significantly less than the number of Phortress-derived DNA adducts detected in MCF-7 xenografts. No adducts were detected in MDA-MB-435 DNA.

**Figure 7:**
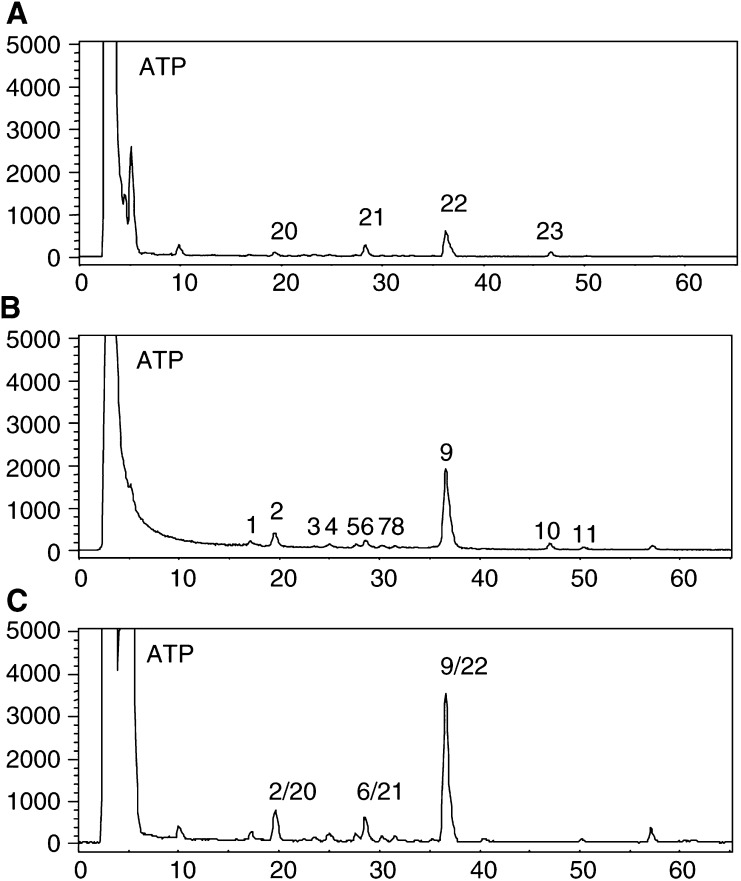
HPLC radiochromatograms of ^32^P-postlabelled adducts. (**A**) DNA (5 *μ*g) from MCF-7 xenograft of Phortress-treated (20 mg kg^−1^, i.p.) mouse. (**B**) DNA (0.5 *μ*g) from MCF-7 cells treated with 1 *μ*M 5F 203 for 72 h. (**C**) Coelution of **A** and **B**.

Data shown in Figure 7

demonstrate that Phortress-derived adducts *in vivo* co-chromatograph with adducts formed by 5F 203 *in vitro*. The retention time of the major adduct detected in MCF-7 tumours of Phortress-treated mice was identical to that seen following treatment of MCF-7 cells *in vitro* with 5F 203. Adducts **20**, **21** and **22** formed in MCF-7 xenografts of mice treated with a single dose of 20 mg kg^−1^ Phortress (24 h) were found to coelute with adducts **2**, **6** and **9** derived from MCF-7 cells exposed to 1 *μ*M 5F 203 (72 h).

**Figure 8:**
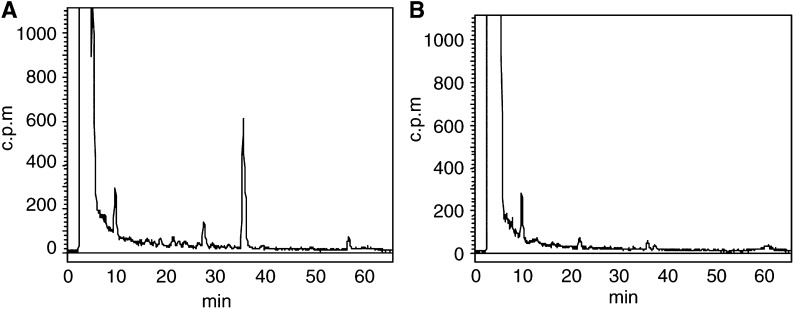
Analysis of DNA adduct formation in tumour xenografts implanted s.c. in opposite flanks of the same mouse recovered 24 h after Phortress treatment (20 mg kg^−1^, i.p.). (**A**) MCF-7. (**B**) MDA-MB-435.

Mice bearing sensitive MCF-7 tumours in one flank and inherently resistant MDA-MB-435 tumours in the opposite flank were treated i.p. with 20 mg kg^−1^ Phortress. After 24 h, animals were killed and tumours were prepared for analysis of DNA adducts. The specificity for DNA adduct formation in sensitive tumours only is unequivocally observed (Figure 8

). Numbers of adducts detected in MCF-7 xenografts did not differ significantly from those previously detected (260±80×10^−8^ nucleotides).

## DISCUSSION

In this study, we have examined the hypothesis that DNA adduct formation may be the biochemical sequel to CYP-mediated metabolic production of putative electrophilic species derived from antitumour 2-(4-aminophenyl)benzothiazoles. It is proposed that the generation of covalent adducts in sensitive cells precedes the exquisitely selective cell death ensuing exposure, *in vitro* and *in vivo*, to analogues of this distinct class of antitumour agent. *In vitro*, sensitivity correlates with induction of CYP1A1 mRNA, activity and protein expression. Engagement of apoptotic machinery (induction of apoptosis-initiating receptor CD95/FAS1, upregulation of P53 and P21, downregulation of Bcl-2; Monks *et al*, 2001; Chua *et al*, 1999a,1999b) and detection of DNA single-strand breaks has been observed in responsive mammary carcinoma cells when challenged with benzothiazole analogues (Bradshaw *et al*, 2000).

Selective generation of DNA adducts in cell lines (MCF-7, IGROV-1) sensitive to 2-(4-aminophenyl)benzothiazole analogues (DF 203, 5F 203, Phortress) has been demonstrated, whereas negligible adduct formation was detected in DNA of inherently resistant HCT 116 colon (Figure 2) and MDA-MB-435 breast carcinoma cells.

Few adducts could be detected in the DNA of sensitive (MCF-7, IGROV-1) cells exposed to concentrations of 5F 203 or Phortress <100 nM. Evidence supports the supposition that DNA repair mechanisms are employed: human damage-specific DNA-binding protein (DDB) mRNA is significantly upregulated in sensitive MCF-7 cells after benzothiazole treatment. DDB, a heterodimer of 48 and 127 kDa subunits, possesses a role in DNA repair and is upregulated in response to UV-induced lesions (Nichols *et al*, 2000). Moreover, DDB activity is absent from a subset of xeroderma pigmentosum (XP) patients possessing a phenotype of defective excision repair (Zolezzi and Linn, 2000). In addition Wip1, a human protein phosphatase induced in response to ionising radiation (Fiscella *et al*, 1997), is also upregulated following benzothiazole treatment. Alternatively, nucleophilic sequestration of low concentrations of reactive intermediates by cellular peptides (e.g. glutathione) or proteins may occur at low drug concentrations. At 1 *μ*M 5F 203 a clear adduct profile emerges in DNA of MCF-7 cells revealing one major and a number of minor adducts. Intriguingly, and emphatically at 10 *μ*M, a new major adduct, **2**, dominates. In MCF-7 cells treated with 10 *μ*M Phortess, the previously minor peak **13**, analogous to the 5F 203-generated peak **2**, becomes an emerging major adduct. This phenomenon fails to occur in IGROV-1 cells whose capacity for adduct burden appears saturated at 1 *μ*M. We may speculate that CYP1A1 and CYP1B1 effect the formation of distinct 5F 203-derived adducts and moreover that CYP1A1- and CYP1B1-induced adducts are generated by exposure to different benzothiazole concentrations. In IGROV-1 cells, CYP1B1 protein expression was not constitutive and only negligibly induced by 5F 203 (1 *μ*M, 10 *μ*M) (Hose *et al*, in press). In MCF-7 cells, constitutively expressed CYP1B1 is further induced by 2-(4-aminophenyl)benzothiazole concentrations exceeding 3 *μ*M concurring with the emergence of major adduct **2** (5F 203) or **13** (Phortress). EROD activity and CYP1A1 expression are maximally induced in cells treated with 1 *μ*M 2-(4-aminophenyl)benzothiazoles concurring with the apparent saturation of adduct **9** (5F 203, MCF-7 cells), **16** (Phortress, MCF-7 cells) or **19** (Phortress, IGROV-1 cells). Indeed, concentrations >1 *μ*M result in irreversible inhibition of CYP1A1 activity (Chua *et al*, 2000).

In V79 cells stably expressing either CYP1A1 or CYP1B1 enzymes, significantly lower concentrations of dibenzo[*a,l*]pyrene are required before the formation of the highly carcinogenic CYP1B1-dependent (−)-anti-dibenzo[*a,l*]pyrene diol epoxide (DB[*a,l*]PDE) adducts (Luch *et al*, 1998). In addition, CYP regioselectivity towards the endogenous hormone 17*β*-oestradiol has been demonstrated; 1A1 catalyses 2-hydroxylation, whereas CYP1B1 is a highly selective oestradiol 4-hydroxylase.

Generation of 5F 203-derived adducts over time (Figure 4) correlated precisely with induction of *CYP1A1* transcription and EROD activity in MCF-7 cells treated with 2-(4-amino-3-methylphenyl)benzothiazoles. *In vitro*, adduct accumulation occurred maximally by 24 h.

Significant DNA adducts were detected in MCF-7 tumour xenografts 24 h after treatment of mice with a concentration of Phortress known to elicit antitumour activity (Bradshaw *et al*, 2002a). Phortress (2×20 mg kg^−1^, i.p.), administered weekly, inhibited the growth of these tumours by 68% (v v^−1^). In IGROV-1 tumours of treated mice, where Phortress administered on the same schedule inhibited xenograft growth by 50%, fewer DNA adduct species were separated; moreover, adducts, whose profile was chromatographically equivalent to that of MCF-7 tumours, were significantly fewer (Figure 6). Negligible adducts were generated in inherently resistant MDA-MB-435 tumours. Thus, adduct burden concurs exactly with the sensitivities of these three xenografts to Phortress. We suggest that in human IGROV-1 xenografts, the major Phortress-derived DNA adduct **24** is equivalent to adduct **22** in MCF-7 tumours. *In vitro*, equivalent Phortress-generated adduct species are represented by peaks **16** and **19** in MCF-7 and IGROV-1 cells, respectively. In MCF-7 cells, Phortress-derived adducts represented by peaks **13** and **16** (Figure 5) are equivalent to 5F 203-generated adducts **2** and **9** (Figure 3). Evidence confirming that a major adduct is commonly induced *in vitro* and *in vivo* by 5F 203 and its lysylamide prodrug has been obtained. DNA extracted from MCF-7 cells exposed to 5F 203 (1 *μ*M, peak **9**) and MCF-7 xenograft tissue of a mouse exposed to Phortress (peak **22**) coelute, demonstrating the generation of identical adduct species.

The exquisite specificity of Phortress-derived adduct generation has been corroborated, following treatment of mice bearing sensitive MCF-7 and inherently resistant MDA-MB-435 tumours in opposite flanks (Figure 8). Thus, tumour sensitivity has been predicted accurately: in antitumour tests, the growth of MCF-7 xenografts was significantly retarded whereas MDA-MB-435 tumours transplanted in the opposite flank continued to grow (Bradshaw *et al*, 2002a). Thus, we propose that the evaluation of DNA adduct formation may provide a valuable pharmacodynamic (PD) end point predictive of tumour sensitivity.

Phortress effects an exquisitely selective antitumour response via a mechanism of action distinct from any clinically used chemotherapeutic agent. It is cleaved in the presence of tumour cells to yield 5F 203, which remains inertly in the milieu of cells immune to this agent. However, once in the presence of a sensitive cancer cell, a cascade of events is initiated resulting in the induction of CYP1A1-catalysed metabolism of 5F 203. Generation of adducts between electrophilic reactive intermediates of 5F 203 and DNA exacts lethal damage that precedes cell death.

**Figure 9:**
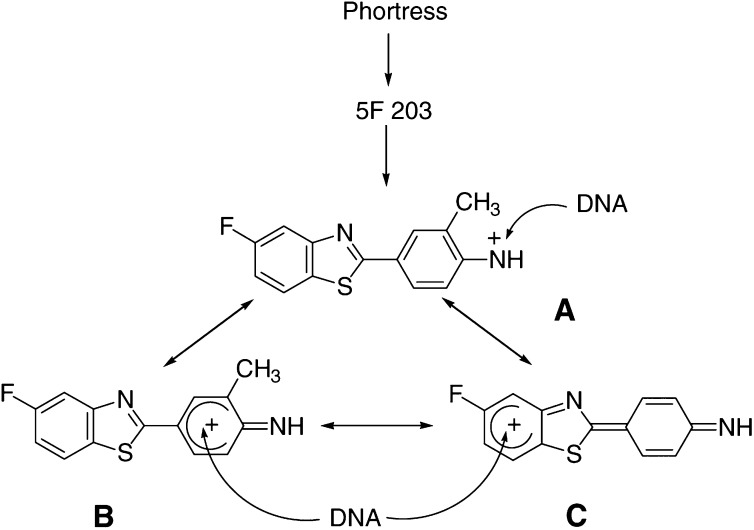
Putative electrophilic reactive intermediates derived from Phortress. Species A is a nitrenium ion. Species B and C are *π*-carbocation mesomeric forms.

FMO calculations predict that a reactive electrophilic nitrenium species may be implicated in the generation of DNA adducts (O'Brien *et al*, in press). Structures of a nitrenium species and *π*-carbocation mesomeric forms derived from 5F 203 are shown in Figure 9

. These structures infer that nucleophilic centres in DNA bases might become adducted at the exocyclic nitrogen (via A), or at carbon atoms in the 2-aryl group (B) or the benzothiazole moiety (C; Stevens *et al*, 1996). This could explain the multiplicity of adducts observed in sensitive tumour cells such as MCF-7 (Figure 3E). We are currently attempting to identify the structures of these adducts to determine why they are so damaging to sensitive tumour cells.

In conclusion, Phortress offers the opportunity for introduction into the clinic of a novel and selective antitumour agent. The techniques described present potential for the measurement of a clearly defined PD end point.

Phortress will undergo clinical evaluation under the auspices of Cancer Research UK and Phase I trials are due to begin in 2003.
